# Hemorrhagic Malarial Fever

**Published:** 1887-07

**Authors:** Benj. H. Riggs

**Affiliations:** Selma, Alabama, Ex-President Medical Association of Alabama


### HEMORRHAGIC MALARIAL FEVER.

By Benj. H. Riggs, M. D., Selma, Ala.,
**Ex-President Medical Association of Alabama.**

Some years ago I became convinced that the pathology of this disease consisted essentially in rapid red-blood destruction, and consequent accumulation of hematin or hemo-globulin in the blood vessels; that the cholemia, the poke-juice urine, and the turgid gall-bladder were due to the systemic efforts for removal of the same from the body. Golding Bird, many years ago, named this hematin or hema-globulin constituents of the urine "purpurine." Holding the above views at that time I named this disease "purpuremia," or "purpurine in the blood." The name did not take because "purpurine" did not hold its own with urinologists; purpurine being hematin in the urine, was called hematin, and now hema-globulin. So my name was buried in the mausoleum of the medical capulets. where it has a populous company; but the more experience I have of the disease the more I am satisfied of the correctness of my theory.

Dr. Michel, of Montgomery, was more fortunate than myself "in getting the ear" of the medical public and got the endorsement of the State Association for his name, "Hemorrhagic Malarial Fever." He says, naming a case, that it "literally bled to death." There I think my good friend was mistaken. I doubt earnestly that any case of "hemorrhagic malarial fever," or "yellow chills," ever bleeds to death. There are many who pass every bit of the blood-coloring matter from their bodies before they die, but rarely, if ever, in the form of blood.

The name, hemorrhagic malarial fever, however, is barely more fortunate than my poor bantling. It is but little known by medical men outside of the South; in England and on the continent it is still less known, and in Senegambia I doubt its ever being heard of. By Northern writers it is called "hematuria," or sometimes "malarial hematuria," by English it is called "hema-globinuria" (a very good name); further east it is called "Fievrejaune hematurique;" and by the people generally it is called "yellow chills."

In volume iv., page 112, of the "System of Medicine," by Pepper, in a scientific article by Dr. James Tyson, of Philadelphia, on kidney diseases, he does not seem to have ever heard of the disease by the name at the head of this article or by any other. He devotes about a page and a half to "malignant malarial hematuria." Just think of it! A man who is first-class authority in the United States on urinary diseases, in such a book as that, ignores all literature on this alarming and common disease! I called Dr. Tyson's attention to this in a note, and he replied asking me to criticise his article. I proposed to do so, and have laid aside some food for an article, but have never yet had time and inclination at the same time to go into it. I will send him a copy of this journal. His article in the above work is entirely inadequate to the dignity of the subject he proposes to handle.

We have had quite a number of these cases in Selma within the last thirty days, and so far as I know nearly all recovered. I have had three to fall to my care. Two in Selma, one a boy about seven years old, and one a young lady about eighteen years old. And one in Froglevel, about six miles from Selma, in a young married woman. All white. They all recovered, and *without any quinine whatever.* The married woman was about four months pregnant, and finally aborted, everything coming away at once. The main reliance in all these cases was arsenic—arsenious acid. I believe that arsenic arrests this blood-destroying process better than any other agent we have. I gave the patient in the country 1-10 grain every three hours until she took ½ grain, and then she took the other half grain in 1-20 grain doses every three and four hours until she took ¾ of a grain. No quinine. The boy took 1-40 grain every three hours until he took ¼ grain. The hematin disappeared promptly in all these cases within the first twelve hours, but the fever continued in the case of the two ladies for several days, and was treated with an alkaline fever mixture and morphine hypodermically. After convalescence I put them all on the sixty-pill prescription of quinine, iron, arsenic, and nux vomica. The set formula that I use in these cases is the following, doses varying according to age and strength of constitution, viz:

℞. Acid. arseniosi .................................. gr. ½,

Piperinæ .................................. gr. ij,

Pulv. Doveri .................................. gr. x,

Ext. hyoscyam .................................. gr. v.

M. and make five capsules. Sig.—One capsule every three hours.

This may be continued until all these are taken, beginning at 6 p. m., and the prescription may be refilled and continued through the next day and night at four hours intervals.

Of course in giving in this way some care must be exercised to prevent too great gastric irrritation or arsenical poisoning. I would advise a careful reading of the article on "Acid Arseniosum" in Wood & Bache's Dispensatory, before any one undertakes to use this treatment.

September 20.—Since writing the above part of this paper, I have had some interesting experience in the treatment of two cases. One ended fatally, and the other is convalescing. The one that died did not really have sufficient nursing attention; or rather, the attention he had was mischievously meddlesome; fatally so, possibly.

Willie F., a boy, white, aged nineteen years, was taken on the evening of September 9th, and died on the afternoon of September 14th. I did not see him until the morning of September 10th, about 7 o'clock; he was then a well-marked case of this disease: fever, cholæmia, distressing nausea of vomiting, and passing quantities of deep mahogany red urine. The urine cleared up that night, Friday, the 10th, and never became discolored any more; but the boy died from exhaustion, from sleeplessness, fever vomiting, etc., on Monday, the 14th. He took the arsenic pill through Friday night in 1-20 grain doses until he had taken five pills, and it was while taking this, that the urine cleared up. What he vomited was alkaline, and his urine was alkaline. Dr. C. J. Clark and myself, carefully examined this urine with a fine microscope, but could not find a trace of a blood corpuscle.

I tested it for bile by the chloroform test, but chloroform coagulated the whole mass by agitation in a test tube. After standing awhile the coagulum settled some to the bottom, leaving a small quantity above of clear pink fluid. On boiling in a test tube nearly fifty per cent. of coagulum was formed. The coagulum formed by the chloroform had a particular bright red look at the thinned margin.

I boiled some of the clear urine on Sunday evening in a spoon, a slight coagulum appeared on the bottom. The specific gravity a specimen taken on Saturday evening was 10.20, normal reaction, natural color, and no albumen.

The other was in a boy, white, aged nine years, now convalescing. This boy passed on three different days in his illness, during the evening exacerbation of the fever, a dark *grayish* or *slate colored* urine, something like weak senna tea. Here was a very unique state of affairs in my experience. He was full of quinine at the time this discharge first appeared, and quinine did not prevent the fever. I gave him Fowler's solution in 3-drop doses that night—the night of my first call—until he took four doses. I reported a case of this disease in the *Medical News*, of Philadelphia, on February 27, 1885. The same young man is now sick with pneumonia, and the first night after my call on him, the pneumonia not being well marked, the spleen enlarged and painful. I gave him 6 drops of liquor Fowleri every three hours, from 9 o'clock at night to 9 o'clock next morning, 30 drops, with no bad effect. His was a reputed case of quinine idiosyncrasy; but his fever being the most prominent feature on yesterday, the temperature being up to 104°, I gave him 20 grains antipyrin at 6 p. m., followed through the night with quinine, bromide, blue mass, and Dover's powders, so that he got 20 grains quinine by 9 a. m., and no bad effects were developed. So that every reputed case of quinine idiosyncrasy is not always such.—*Alabama Medical and Surgical Journal.*

